# Supplementary material for: A novel de novo FEM1C variant is linked to neurodevelopmental disorder with absent speech, pyramidal signs and limb ataxia
Source: Hum Mol Genet. 2022 Nov 7;32(7):1152–61. doi: 10.1093/hmg/ddac276 (PMC10026218; doi:10.1093/hmg/ddac276)
Supplement: Supp_legend_ddac276 [file supp_legend_ddac276.docx]

**Supplementary Figure**

**A.** Sequencing chromatogram of CRISPR/Cas9-generated FEM-1^Asp133His^ mutation displaying sequence peaks and base calls. Data was visualized in the SnapGene 6 software (Insightful Science; snapgene.com). **B.** Number of unhatched eggs counted after 16 hours of laying for wild-type (*n*=95) and FEM-1^Asp133His^ (*n=*92) worms; *N*=5*. n* represents the total number of worms; *N* represents the number of experimental repeats. The stars denote the level of significance of the *p*-value obtained by the Mann–Whitney test. *p*-value is shown adjacent to the graph (****p* ≤ 0.001). Data was plotted and analyzed in the GraphPad Prism 9 software. **C.** Egg-laying assay of wild-type (*n=95*) and FEM-1^Asp133His^ (*n*=92) worms; *N=5*. *n* represents the total number of worms; *N* represents the number of experimental repeats. *p*-value was calculated obtained by the Mann–Whitney test and is shown adjacent to the graph (ns - not significant). Data was plotted and analyzed in the GraphPad Prism 9 software. **D.** Illustration of aldicarb’s and levamisole's mechanism of action. The transmission of impulses between neurons is mediated by the synthesis of Ach (magenta star) at the presynapse and its release into the synaptic cleft, where it binds to Ach receptors (only nAchR are shown). Aldicarb (depicted as a stop sign) inhibits AchE (navy pac-man), resulting in the build-up of Ach, which causes muscle paralysis. Levamisole (yellow star) is an allosteric modulator of nAchR, and its binding to this receptor assures the continuity of the action potential, resulting in muscle contraction and paralysis. Graphic created with BioRender.com. **E.** Levamisole sensitivity assay of wild-type (*n*=129) and FEM-1^Asp133His^ (*n*=122) worms; *N*=3. *n* represents the total number of worms; *N* represents the number of experimental repeats. *p*-value was calculated using the Log-rank (Mantel-Cox) test and is shown adjacent to the graph (ns - not significant). Data was plotted and analyzed in the GraphPad Prism 9 software.
