# Supplementary figures and images for: A novel de novo FEM1C variant is linked to neurodevelopmental disorder with absent speech, pyramidal signs and limb ataxia
Source: Hum Mol Genet. 2022 Nov 7;32(7):1152–61. doi: 10.1093/hmg/ddac276 (PMC10026218; doi:10.1093/hmg/ddac276)

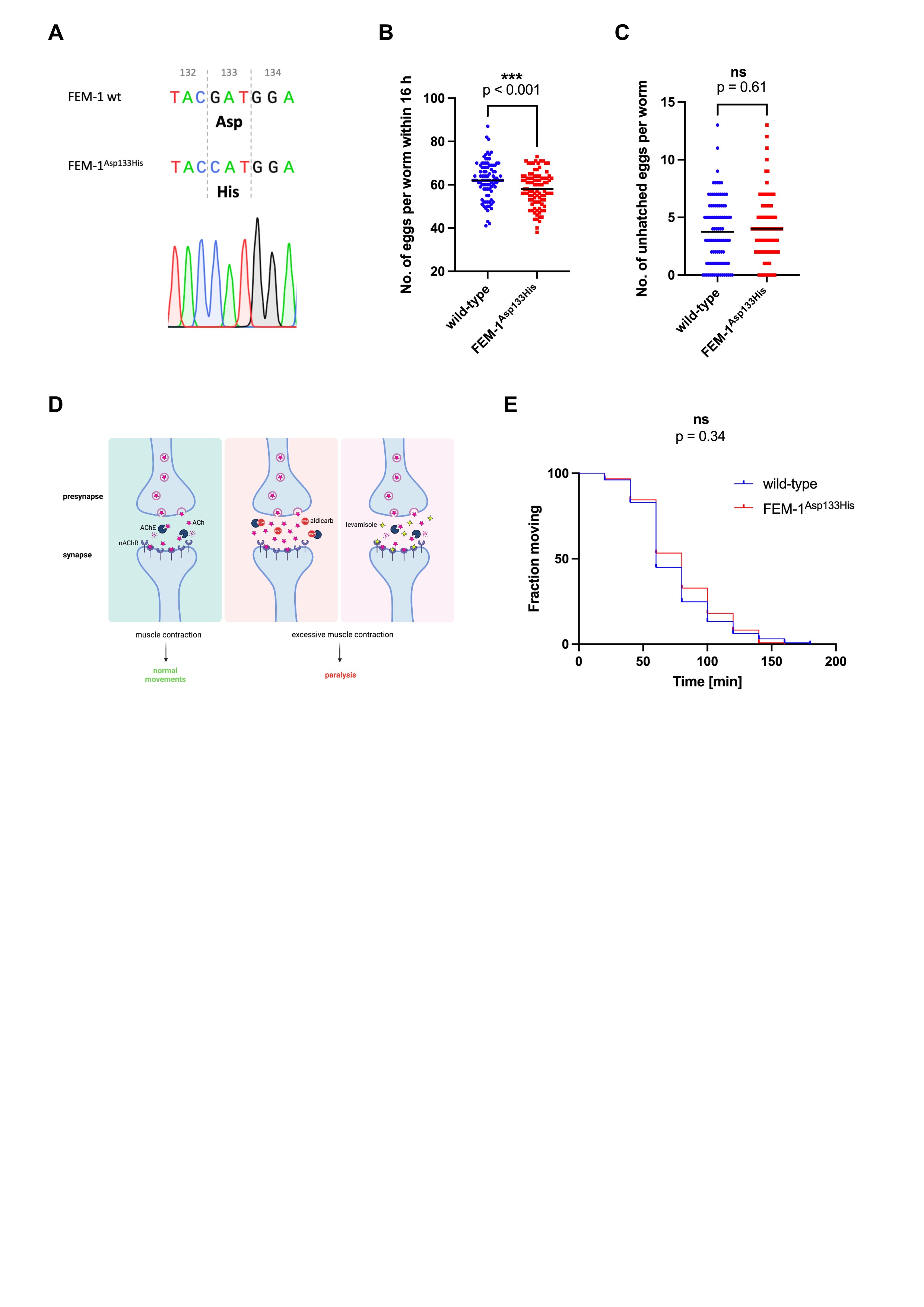

Supplement: Sup_ddac276 [file sup_ddac276.jpeg]
